# Quadricipital Tendon Simultaneous Bilateral Tear in a Patient with Type-II Diabetes Mellitus after Low-energy Trauma: Case Report *

**DOI:** 10.1055/s-0040-1722584

**Published:** 2021-03-31

**Authors:** Breno Almeida de Pinho Tavares, Leonardo Cortes Antunes, Sara Johanna Sanchez Guerrero, Ângelo José Nascif de Faria, Renato Amorim Carvalho, Rademack Duarte Amorim

**Affiliations:** 1 Hospital São Francisco de Assis, Belo Horizonte, MG, Brasil; 2 Departamento de Ortopedia e Traumatologia, Hospital São Francisco de Assis, Belo Horizonte, MG, Brasil; 3 Hospital Santa Lúcia Norte, Brasília, DF, Brasil; 4 Prontomed, Teresina, PI, Brasil

**Keywords:** knee, quadriceps muscle, tendon injuries, rupture

## Abstract

Simultaneous bilateral rupture of the quadricipital tendon is an extremely rare lesion. We report a case of this injury after low-energy trauma in a patient with type-II diabetes mellitus. Both knees were surgically approached in the same surgical procedure. Early rehabilitation is essential for the adequate functional recovery of the knee. The aim of the present report was to describe an atypical case of this type of injury after minimal trauma, as well as to detail the surgical technique used to treat it.

## Introduction

Total rupture of the quadriceps tendon is a well-known entity in orthopedics that requires surgical treatment to restore extensor function. 1 This injury is a rare condition. 2 First described by Steiner and Palmer 3 in 1949, the lesion is most commonly found in patients older than 50 years of age. There have been cases of this lesion described that were related to previous pathologies, such as rheumatoid arthritis, systemic lupus erythematosus, arteriosclerosis, diabetes mellitus (DM), primary and secondary hyperparathyroidism, gout, tuberculosis and vasculitis, 4 with chronic renal failure (CRF) being the most common risk factor found in these ruptures. 5

## Case Report

A 66-year-old male patient complained of acute pain in his knees and functional incapacity. He reported that as he was going down a flight of stairs, he jumped to the ground, fell with his feet supported and his knees bent, suddenly felt bilateral pain at the level of the knee, and was unable to walk. Upon physical examination, we could observe bilateral joint effusion (++/4+), the presence of a gap in a region of the upper pole of the patella in the right knee, and a gap in the quadriciptal myotendinous transition in the left knee. He was asked to perform, separately, active elevation of the lower limbs in extension, but he couldn't maintain this position against gravity. Radiographs showed bilateral suprapatellar syndesmophyte and inferiorized kneecap. In the previous history of the patient, there was a report of controlled diabetes mellitus using oral antiglycemic agents. His weight was within normal range according to the body mass index (BMI). The laboratory tests were normal.

**Fig. 1:**
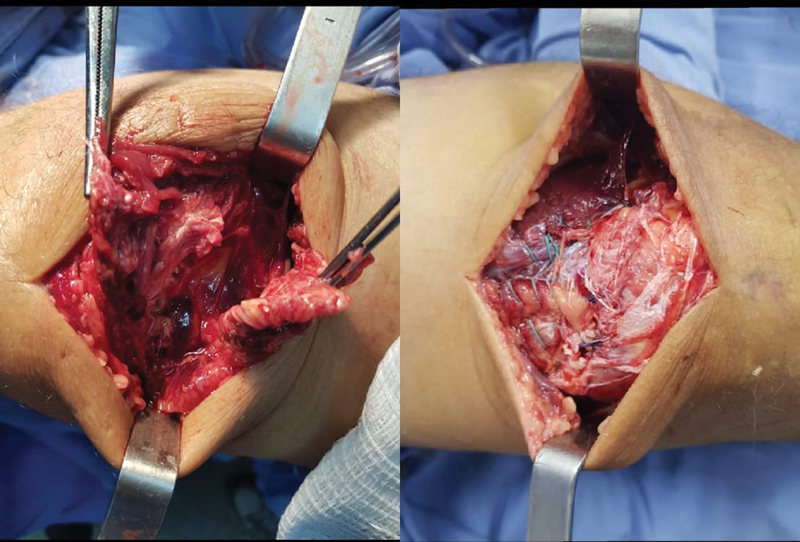
Quadricipital tendon injury of the right knee and radiographic image of the postoperative period.

**Fig. 2:**
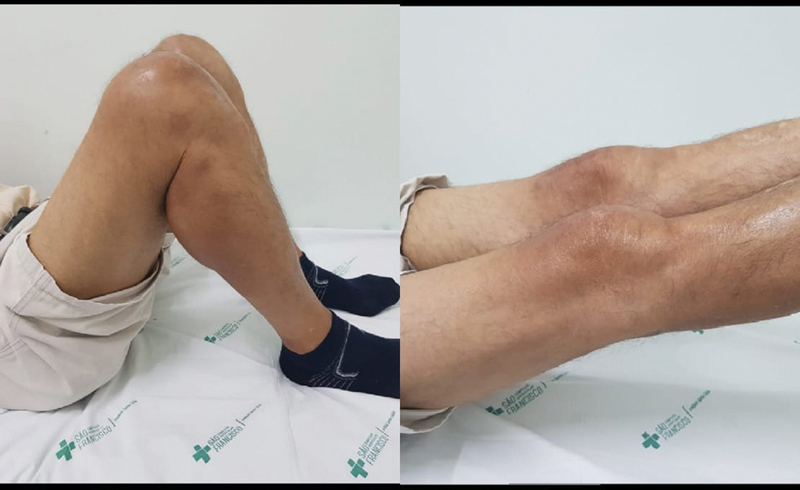
Quadricipital tendon injury of the left knee and termino-terminal suture of the quadriceps tendon.

The patient underwent surgical treatment two days after the injury. Both knees were approached in the same surgical procedure. Under spinal anesthesia and with the use of two tourniquets at the root of the thighs (inflated separately), we performed an anterior median incision of about 7 cm, from distal to the proximal, in the upper pole of the patella in the right knee ( Fig. 1 ). After surgical exploration, complete disinsertion of the quadriceps tendon was identified in the proximal region of the kneecap. After hematoma removal, repair of the stump proximal to the upper kneecap pole was performed using the Krackow 6 technique with nonabsorbable number-2 wires (Ethibond, Medline Industries, Inc., Northfield, IL, US). We also used two 5-mm anchors in the upper pole of the patella, thus performing the tendon reinsertion, with the knee at 40° of flexion. The left knee injury was approached with a similar incision: a complete lesion was observed in the myotendinous junction of the quadriceps tendon, and the termino-terminal suture was performed using the the Krackow 6 technique with Ethibond number-2 wires, with the knee at 40° of flexion ( Fig. 2 ). In both knees the retinacular lesions were reassembled with Vycril 2.0 (Ethicon, Somerville, NJ, US).

In the immediate postoperative period, the knees were immobilized with plastered splints for three weeks. Seven days after surgery, isometric quadriceps exercises were initiated. The rehabilitation with self-passive physiotherapy evolved with satisfactory range of motion. Load with the aid of a walker was allowed within six weeks of evolution.

## Discussion

According to Kellersmann et al., 2 this is a rare entity, usually related to the presence of chronic systemic diseases or steroid use. The most common risk factor is CRF, as reported by Tomazini et al. 7 According to Kara et al., 4 the most common mechanism of trauma is the sudden and eccentric contraction of the quadriceps muscle, with knee flexion concomitant with the feet fixed to the ground.

According to Matokovic et al., 5 DM is responsible for functional and structural changes in both macrocirculation and tendon microcirculation, resulting in biochemical and structural abnormalities in various organs and tissues, including the tendons. The work of Sharma and Maffulli 8 showed that the endothelial lesions promoted by DM cause a reduction in the synthesis and secretion of protective factors leading to changes that provide a proconstrictive, proinflammatory and proaggregating state to the blood vessel, generating a decrease in blood supply. We believe that this is the main explanation for the tendon rupture described.

For Ilan et al., 9 the diagnosis of this lesion is based on the clinical findings. Upon physical examination, the patient usually has a palpable gap above the patella, and the active extension of the knees is compromised. Other signs include hemarthrosis, absence of patellar reflexes, or presence of floating patella. These signs and symptoms suggest the diagnosis of the lesion. According to Kim et al., 10 radiographs of the knees should be requested; however, they may show only some indirect signs of rupture. In cases of diagnostic doubt or associated lesions, magnetic resonance imaging and computed tomography may be useful. Cases of complete rupture require surgical treatment, and early surgical intervention is a determining factor for a good outcome. Late surgical repair has been associated with worse functional outcomes. Most patients with simultaneous bilateral quadriceps tendon repairs can expect a good functional outcome; however, many will have difficulty returning to high-level sports activities.

**Fig. 3:**
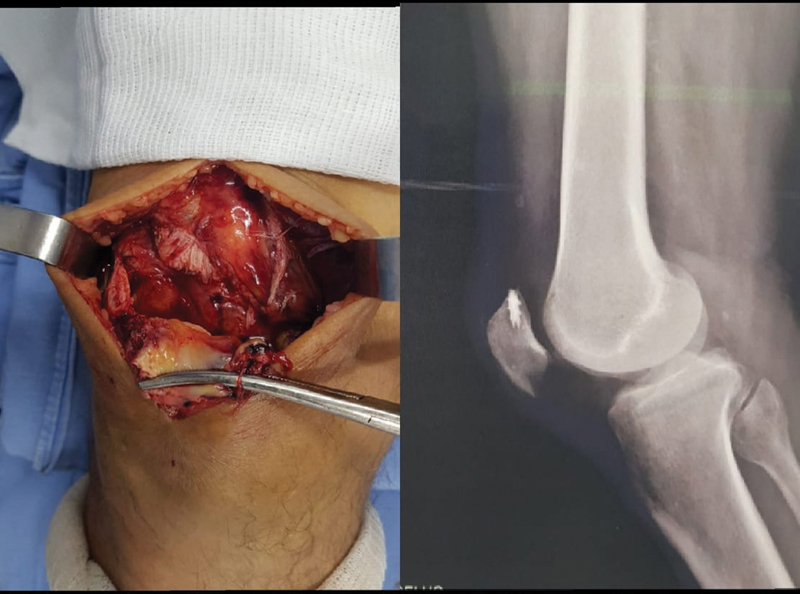
Patient three months postoperatively presenting good range of motion and preserved function of the extensor mechanism bilaterally.

The orthopedic literature is scarce regarding reports of simultaneous bilateral quadricipital tendon rupture in a patient with DM. The authors of the present study reported a rare case in which the patient, whose underlying disease was type-II DM, suffered this injury after minimal trauma. It is believed that this chronic disease is an important cause of weakening of the structure of the quadriciptal tendon, whose main reason for rupture would be the reduction of local blood supply due to changes in its micro and macro circulation. The bilateral nature of the lesion can hinder rehabilitation. The main reasons for the excellent functional results include early tendon repair with adequate suture tension and strength, reduced immobilization time, and an effective physiotherapy program ( Fig. 3 ).
