# Supplementary material for: Quadricipital Tendon Simultaneous Bilateral Tear in a Patient with Type-II Diabetes Mellitus after Low-energy Trauma: Case Report
Source: Rev Bras Ortop (Sao Paulo). 2021 Mar 31;59(1):e136–8. doi: 10.1055/s-0040-1722584 (PMC10957280; doi:10.1055/s-0040-1722584)
Supplement: Related texts

# Research article: Ruptura bilateral simultânea do tendão quadricipital em paciente portador de diabetes mellitus do tipo II após trauma de baixa energia: Relato de caso *

**Keywords:** joelho, músculo quadríceps, traumatismos dos tendões, ruptura

## Abstract

A ruptura bilateral simultânea do tendão do quadríceps é uma lesão extremamente rara. Relatamos um caso desta lesão após trauma de baixa energia em paciente portador de diabetes mellitus do tipo II. O diagnóstico é essencialmente clínico, e requer tratamento precoce. Os dois joelhos foram abordados cirurgicamente no mesmo ato operatório. A reabilitação precoce é fundamental para a recuperação funcional adequada do joelho. O objetivo deste relato foi descrever um caso atípico deste tipo de lesão após trauma mínimo, bem como detalhar a técnica cirúrgica utilizada para o tratamento.

## Introdução

A ruptura total do tendão do quadríceps é uma entidade bastante conhecida na ortopedia, e necessita de tratamento cirúrgico para o restabelecimento da função do mecanismo extensor. 1 Esta lesão é uma condição rara. 2 Descrita pela primeira vez por Steiner e Palmer 3 em 1949, é mais comumente encontrada em pacientes maiores de 50 anos. Foram descritos casos desta lesão relacionados a patologias prévias, como artrite reumatoide, lúpus eritematoso sistêmico, arteriosclerose, diabetes mellitus (DM), hiperparatireoidismo primário e secundário, gota, tuberculose e vasculites, 4 sendo a insuficiência renal crônica (IRC) o fator de risco mais encontrado nessas rupturas. 5

## Relato de Caso

Paciente do sexo masculino, 66 anos, que apresentava queixa de dor aguda nos joelhos e inpacidade funcional. Ele relatou que, ao descer uma escada, realizou um salto ao solo, caiu com os pés apoiados e os joelhos fletidos, sentiu subitamente dor bilateral no nível do joelho, e não conseguiu deambular. Ao exame físico, observou-se derrame articular (++/4 + ) bilateral, presença de hiato na região do polo superior da patela no joelho direito, e na transição miotendinosa quadriciptal no esquerdo. Solicitou-se que realizasse, separadamente, elevação ativa dos membros inferiores em extensão, mas ele não conseguiu manter a posição contra a gravidade. Radiografias evidenciaram sindesmófito suprapatelar bilateral e rótula inferiorizada. No histórico do paciente, havia um relato de DM controlada com o uso de antiglicêmicos orais. Seu peso encontrava-se dentro da normalidade segundo o índice de massa corporal (IMC), e os exames laboratoriais apresentavam-se normais.

O paciente foi submetido a tratamento cirúrgico dois dias após a lesão. Os dois joelhos foram abordados no mesmo ato operatório. Sob raquianestesia e com o uso de dois torniquetes (inflados separadamente) na raiz das coxas, foi realizada incisão mediana anterior de cerca de 7 cm de distal para proximal a partir do polo superior da patela no joelho direito ( Fig. 1 ). Após a exploracão cirúrgica, foi identificada desinserção completa do tendão do quadríceps na região proximal da rótula. Após a retirada do hematoma, foi realizado reparo do coto proximal ao polo superior da rótula com a técnica de Krackow, 6 utilizando fios inabsorvíveis número 2 (Ethibond, Medline Industries, Inc., Northfield, IL, EUA). Também foram utilizadas 2 âncoras de 5 mm no polo superior da patela, realizando-se dessa forma a reinserção tendinosa, com o joelho em 40° de flexão. A lesão do joelho esquerdo foi abordada com incisão semelhante, sendo observada uma lesão completa na junção miotendinosa do tendão do quadríceps e realizada a sutura término-terminal pela técnica de Krackow, 6 utilizando fios Ethibond número 2, com o joelho em 40° de flexão ( Fig. 2 ). Em ambos, os joelhos as lesões retinaculares foram reparadas com Vycril 2.0 (Ethicon, Somerville, NJ, EUA).

**Fig. 1:** Lesão do tendão quadriciptal do joelho direito e imagem radiográfica do pós-operatório.

**Fig. 2:** Lesão do tendão quadriciptal do joelho esquerdo e sutura término-terminal do tendão do quadríceps.

No pós-operatório imediato, os joelhos foram imobilizados com talas gessadas por três semanas. Sete dias após a cirurgia, foram iniciados os exercícios isométricos do quadríceps. A reabilitação com fisioterapia autopassiva evoluiu com amplitude de movimento satisfatória. A carga com auxílio de andador foi permitida com seis semanas de evolução.

## Discussão

Segundo Kellersmann et al., 2 esta é uma entidade rara, geralmente relacionada à presença de doenças sistêmicas crônicas, ou ao uso de esteroides. O fator de risco mais encontrado é a IRC, conforme relataram Tomazini et al. 7 De acordo com Kara et al,. 4 o mecanismo de trauma mais comum é a contração súbita e excêntrica do músculo quadríceps com flexão do joelho concomitante aos pés fixos ao solo.

Segundo Matokovic et al., 5 a DM é responsável por alterações funcionais e estruturais tanto na macrocirculação quanto na microcirculação tendinosa, acarretando anormalidades bioquímicas e estruturais em vários orgãos e tecidos, inclusive nos tendões. O trabalho de Sharma e Maffulli 8 mostrou que as lesões endoteliais promovidas pela DM causam uma redução na síntese e secreção dos fatores protetores, levando às alterações que propiciam um estado pró-constritor, pró-inflamatório e pró-agregante ao vaso sanguíneo, gerando uma diminuição de aporte sanguíneo. Acreditamos que esta seja a principal explicação para a ruptura tendinosa descrita.

Para Ilan et al., 9 o diagnóstico desta lesão é baseado em achados clínicos. Ao exame físico, o paciente geralmente apresenta um hiato palpável acima da patela, e a extensão ativa dos joelhos fica comprometida. Outros sinais incluem a hemartrose, a ausência de reflexos patelares, ou a presença de patela flutuante. Esses sinais e sintomas sugerem o diagnóstico da lesão. De acordo com Kim et al., 10 as radiografias dos joelhos devem ser solicitadas; no entanto, podem mostrar apenas alguns sinais indiretos de ruptura. Em casos de dúvida diagnóstica ou lesões associadas, a ressonância magnética e a tomografia computadorizada podem ser úteis. As rupturas completas requerem tratamento cirúrgico, sendo a intervenção cirúrgica precoce um fator determinante para um bom resultado. O reparo cirúrgico tardio tem sido associado a piores resultados funcionais. A maioria dos pacientes com reparos bilaterais do tendão do quadríceps simultâneos podem esperar um bom resultado funcional; entretanto, muitos apresentarão dificuldade para retornar às atividades esportivas de alto nível.

A literatura ortopédica é escassa quanto aos relatos de ruptura bilateral simultânea do tendão quadricipital em paciente portador de DM. Os autores do presente estudo relataram um caso raro em que o paciente sofreu esta lesão após um trauma mínimo, cuja doença de base era a DM do tipo II. Acredita-se que essa doença crônica seja uma causa importante de enfraquecimento da estrutura tendinosa quadriciptal, cujo principal motivo de ruptura seria a redução do aporte sanguíneo local devido às alterações na sua micro e macrocirculação. A natureza bilateral da lesão pode dificultar a reabilitação. As principais razões para os excelentes resultados funcionais incluem: reparo precoce do tendão com a tensão e força adequadas da sutura, o tempo reduzido de imobilização, e um programa de fisioterapia eficaz ( Fig. 3 ).

**Fig. 3:** Paciente com três meses de pós-operatório apresentando boa amplitude de movimento e função preservada do mecanismo extensor bilateralmente.
